# P4 Reproductive Medicine: Prediction, Prevention, Personalization, and Participation in Infertility Care

**DOI:** 10.3390/jcm13195860

**Published:** 2024-10-01

**Authors:** Danilo Cimadomo, Andrea Garolla, Amerigo Vitagliano

**Affiliations:** 1 IVIRMA Global Research Alliance, Genera, Clinica Valle Giulia, 00197 Rome, Italy; 2 Unit of Andrology and Reproductive Medicine, Department of Medicine, University of Padova, 35100 Padova, Italy; andrea.garolla@unipd.it; 3 Unit of Obstetrics and Gynecology, Department of Interdisciplinary Medicine, University of Bari, 70124 Bari, Italy; amerigo.vitagliano@gmail.com

Infertility affects approximately 10–15% of couples in their reproductive age, and its impact is escalating globally. The IFFS has recently highlighted how fertility rates have dropped below replacement levels in many countries, with growing concern about population decline and its economic, social, and cultural impacts. The experts have emphasized the importance of family building policies, including access to fertility care, as crucial responses to these trends. Equitable access to reproductive technologies and family friendly policies are urgently needed to support individuals and couples in achieving their desired family sizes [1]. As reproductive medicine evolves, the integration of new therapies, advanced technologies, and personalized care approaches is reshaping the field, driving us toward P4 Reproductive Medicine—prediction, prevention, personalization, and participation. This Special Issue embraces some of the most recent findings in this scenario, highlighting how these four pillars are transforming infertility care and optimizing patient outcomes.

## 1. Integrative and Multidisciplinary Approaches in Reproductive Medicine

Embracing P4 Reproductive Medicine means establishing a multidisciplinary team that can support the couples from several complementary perspectives throughout their journey. In this Special Issue, the paper entitled “Before Is Better: Innovative Multidisciplinary Preconception Care in Different Clinical Contexts” highlights the importance of this integrative approach [contribution 1]. The proposed model of preconception care includes collaboration among gynecologists, andrologists, geneticists, psychologists, and nutritionists, emphasizing the need to tailor the approaches to each couple’s unique medical and reproductive history. The main goal is to identify advances and to mitigate any potential reproductive risks, thereby improving maternal and fetal outcomes and reinforcing the role of comprehensive, team-based strategies in enhancing reproductive health.

## 2. Prediction: Enhancing Decision-Making with AI

Prediction plays a crucial role in reproductive medicine, enabling personalized care through data-driven insights. A multicenter study demonstrated how AI/ML-driven prognostic tools—in this Special Issue, the Univfy^®^ PreIVF Report was tested—may support IVF utilization by providing personalized and trustworthy live birth probabilities tailored to individual couples’ data and fertility center specifics. Yao and colleagues showed how the tool increased IVF conversion rates and empowered patients’ decision-making with precise predictions of their treatment success [contribution 2]. By integrating predictive analytics, reproductive medicine is moving towards a future where clinicians can offer patients not just a chance, but a scientifically validated likelihood of success; this scenario is extremely valuable to sustain the comeback of a multicycle approach in reproductive care [2,3]. This approach aligns with the principle of prediction, making ART more efficient and accessible by tailoring treatments to the patient’s unique circumstances. In the context of embryo selection, another study that is part of this Special Issue tested an AI tool named iDAScore v1.0, a deep-learning model designed to grade embryos based on time-lapse videos with no need for human intervention. The iDAScore showed a positive association with embryos’ chromosomal and reproductive competence; a clinical simulation also indicated that the tool would have prioritized euploid embryos for transfer—in the presence of aneuploid from the same cohort—in 60% of the cases, and that it would have disagreed on euploid blastocysts’ ranking priority for transfer in 50% of the cases [contribution 3]. This divergence of opinion with respect to the embryologists’ view outlines the room for improvement of AI applications in relation to embryo selection effectiveness. A recent multicenter RCT suggested that iDAScore can at least match the clinical outcomes of traditional manual embryologists’ assessments, while significantly reducing the time required for embryo evaluation [4]. AI is entering IVF clinics, and it will revolutionize reproductive care from pre-IVF counseling down to embryo transfer. In this perspective, a careful process of validation and implementation will foster the achievements of a plateau of productivity in the coming years.

## 3. Prevention and Personalization: Tailoring Treatment Protocols to Individual Needs and Mitigating Risks in IVF

Prevention is a cornerstone of P4 Reproductive Medicine, focusing on reducing the risks associated with infertility treatments. A study in this Special Issue investigated the role of low-dose aspirin in programmed cryopreserved embryo transfer cycles. Despite its widespread use due to its anti-inflammatory properties, aspirin did not significantly improve live birth rates or reduce complications like hypertension disorders of pregnancy or gestational diabetes mellitus [contribution 4]. This finding adds evidence regarding the ongoing controversy of administering adjuncts to improve transfer success or obstetrical outcomes [5], highlighting the importance of targeted preventive measures and demonstrating that not all interventions are universally beneficial. In general, it is key to identify which patients might benefit from specific therapies, rather than adopting a one-size-fits-all approach. Personalization should balance benefits and potential risks based on each patient’s unique profile. For instance, repeated implantation failure (RIF) is a matter of concern in IVF, as it raises doubts in terms of definition, and consequently diagnosis and treatment. ESHRE has recently discussed this topic in two official documents [5,6], and recent evidence questions whether idiopathic RIF is a condition per se or just an epiphenomenon of IVF [7,8]. A retrospective cohort study that was part of this Special Issue explored the results of autologous leukocyte-poor platelet-rich plasma (LP-PRP) infusion in RIF patients (whose definition did not fulfill either ESHRE 2023 good practice recommendations or the Lugano consensus). The study showed promising results in line with the literature on this topic [contribution 5]. Nevertheless, an RCT is now needed in a better-defined population of RIF women, and possibly in the context of euploid blastocyst transfers. The future of IVF and of add-on implementation demand a careful profiling of the patients who might benefit from them guided by evidence-based criteria. 

## 4. Participation: Reassuring Data to Support IVF Patients in Their Reproductive Journey

Participation is essential across a journey full of hurdles and threatened by misinformation and fake news. Patients eagerly need to be reassured and provided with valuable and reliable information. In this scenario, the multicenter retrospective study by Hervás and colleagues included in this Special Issue demonstrates that elevated sperm DNA fragmentation in IVF–ICSI treatments does not significantly impact pregnancy or neonatal outcomes [contribution 6]. Their findings help dispel common misconceptions, emphasizing that patients can participate confidently in ART without undue concern over paternal DNA damage. The clear communication of results like these is crucial in reducing anxiety and supporting informed, participatory decision-making in reproductive care.

## 5. Conclusions

The convergence of predictive analytics, preventive strategies, personalized treatment protocols, and participatory patient care is setting a new standard in reproductive medicine. By embracing the principles of P4 Reproductive Medicine, we are not only improving the success rates of fertility treatments, but also enhancing the overall patient experience. As the field continues to evolve, the focus must remain on leveraging advanced technologies and individualized care to support patients in their reproductive journeys, ultimately achieving the best possible outcomes with minimal risks and stress. The future of infertility care is bright, grounded in a holistic, patient-centered model that prioritizes the unique needs and aspirations of every couple seeking to build their family.
